# Utilizing Recyclable Task-Specific Ionic Liquid for Selective Leaching and Refining of Scandium from Bauxite Residue

**DOI:** 10.3390/molecules26040818

**Published:** 2021-02-04

**Authors:** Eleni Mikeli, Efthimios Balomenos, Dimitrios Panias

**Affiliations:** 1 Laboratory of Metallurgy, National Technical University of Athens, Zografos Campus, 15780 Zografos, Greece; efthymios.balomenos-external@alhellas.gr; 2 Mytilineos SA-Metallurgy Business Unit, Alumina and Aluminium Plant, Ag Nickolas, 32003 Viotia, Greece

**Keywords:** ionic liquids, scandium, bauxite residue, IL losses

## Abstract

Ionic liquids (ILs) have attracted great interest in the field of extractive metallurgy mainly because they can be utilized in low temperature leaching processes where they exhibit selectivity and recyclability. A major drawback in mixed aqueous-IL systems, is IL dissolution in the aqueous phase, which leads to IL losses, increasing the overall processing cost. This study advances the method for recovering scandium (Sc) from bauxite residue (BR) using as leaching agent the IL betainium bistriflimide, [Hbet][Tf_2_N] mixed with water, which has been reported in previous publications. Ionic liquid leachate (IL-PLS) was prepared by leaching BR with a mixture of [Hbet][Tf_2_N]-H_2_O and subjected to different stripping experiments using hydrochloric acid. The advancement, presented in this work, is related with the optimization of the metal extraction (stripping) from the IL-PLS, where an aqueous solution with high Sc concentration and minimum metal impurities and minimum IL co-extraction is produced. It is further proven that the metal cation extraction is defined by the stoichiometry of the acidic solution and the dissolution (losses) of the IL in the aqueous phase can be minimized by adjusting the volume ratio and the acid concentration. A two-step stripping process described, achieves the selective increase of Sc concentration by 8 times in the aqueous solution, while maintaining cumulative IL losses to similar levels as the optimum 1 step non-Sc selective stripping process.

## 1. Introduction

Ionic liquids (ILs) are described as molten salts, consisting solely of an organic cation and an inorganic or organic anion. Due to the bulky, asymmetrical structure of the constituent ions and their high ionic interaction, ILs can remain in liquid state at significantly lower temperatures compared to typical salts which have high melting points (>800 °C) [1]. One of the most promising aspects of ILs is that their properties can be tailored by modifying their structure, such as the combination of anions/cations pairs, the alkyl chain length and the attached functionalized alkyl group. During the last decades, ILs have been investigated for different applications, mainly due to their potential use in low temperature processes. Until now they have already been applied in larger scales in industrial IL-assisted processes, as solvents, electrolytes, catalysts, performance additives etc. [2].

The idea that ILs could replace typical industrial solvents which are generally considered toxic, volatile and flammable, has aroused great interest in the field of metallurgy. There is a significant group of ILs that are partially or conditionally immiscible with water and have been examined in many studies as media for metal extraction, either in metal oxides dissolution, or in liquid/liquid separation processes [3,4]. An important issue in such process is controlling the mutual solubility of IL-H_2_O systems. Even the most hydrophobic ILs exhibit a non-negligible mutual-solubility with water, [5,6,7,8], resulting in their significant losses when mixed with aqueous phases, during metallurgical processes.

An illustrative example of these ILs is the betainium bis(trifluoromethylsulonyl) imide, [Hbet][Tf_2_N]. This IL is a weak Brønsted acidic IL due to the carboxyl group attached to its cation, [Hbet]^+^, that is known to coordinate with various metal ions [9]. Since its development from Nockemann et al. in 2006 [10], [Hbet][Tf_2_N], has been extensively investigated in different metallurgical processes for dissolution and/or extraction of Sc, REEs [11,12,13,14,15,16,17,18], In [19,20], Th [20], uranyl species [21,22,23], Au [24] and PGMs [25,26].

Even though [Hbet][Tf_2_N] is classified as a hydrophobic IL, it is known to exhibit partial miscibility with aqueous solutions. The dissolution of the IL in the final aqueous streams raises concerns regarding its wider use in larger scales. Increased losses of the IL can lead to a large increase in the cost of a designed process, making it non-viable for industrial use. In addition, high concentration of IL can lead to organic contamination in the final solution making it difficult to process it downstream. This problem has not been addressed yet, as most works that use [Hbet][Tf_2_N] fail to report this problem and focus only on the initial leaching step. Moreover, the behavior of the IL upon contact with different aqueous systems is not fully described yet.

In our previous publications [11,12], we have successfully utilized the IL [Hbet][Tf_2_N] as a leaching agent for BR. BR is the solid by-product of Bayer process for alumina production and in many cases has considerably high concentration of Sc (90–100 ppm). The IL [Hbet][Tf_2_N] has a temperature dependent phase behavior, also called thermomorphism [10]. Below a specific temperature, which is reported as the Upper Critical Solution Temperature (UCST), [Hbet][Tf_2_N]-H_2_O system is a two-phase mixture, while above that temperature the IL form homogenous solutions with water. In the process described in our previous work, the selective dissolution of metals from BR can be achieved by using mixture of [Hbet][Tf_2_N]-H_2_O at temperatures above the UCST, where the mixture is a homogenous acidic solution. Metals dissolved into [Hbet][Tf_2_N] can be extracted in a subsequent stripping step with an acidic solution at ambient temperature regenerating the IL which is separated from the aqueous solution phase for reuse. Extraction of Sc from BR with [Hbet][Tf_2_N], is highly selective against Fe which is the most abundant element in BR. Moreover, due to the organic composition of the [Hbet][Tf_2_N]-H_2_O mixtures, no silica-gel formation is observed, making this IL a promising substitute of conventional mineral acids.

**Figure 1:**
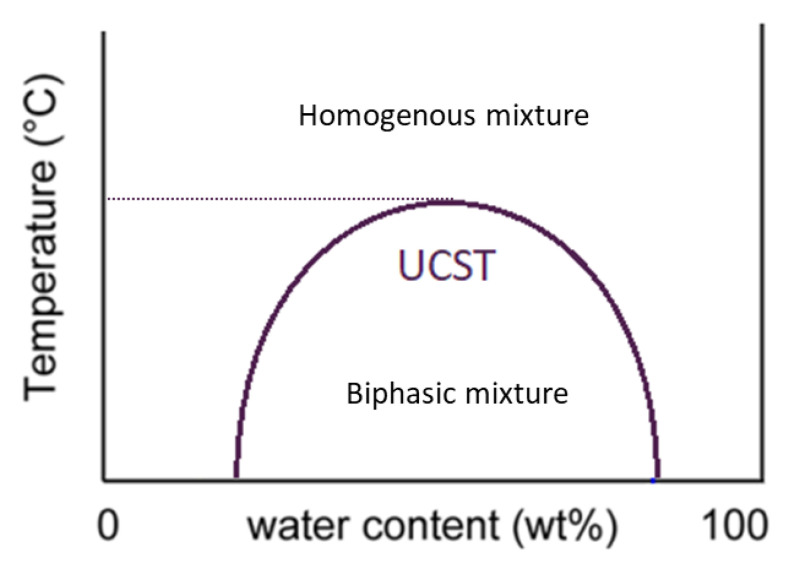
Schematic overview of liquid-liquid phase diagram of IL/water systems with a UCST.

In order to determine the losses of the IL in the aqueous strip solution and to optimize the extraction processes a detailed understanding of the phase behavior between aqueous phases and IL system is required [27]. Mixtures that present UCST have a distinctly shaped liquid-liquid phase diagram, shown in Figure 1. From this diagram, the mutual solubility of the solvents, can be determined.

In the study of Nockemann et al., the phase diagram of [Hbet][Tf_2_N]-H_2_O was identified experimentally [10], the solubility of [Hbet][Tf_2_N] in the water phase was measured to be 14%wt, and the UCST reported to be 55 °C, values that were later confirmed by other studies too [14]. According to following studies, it is now known that these values can only refer to pure IL-water mixtures, as the properties of the ILs can significantly change during metal dissolution [14]. In the study of Fagnant et al [7], it was reported that metal loading of [Hbet][Tf_2_N] can shift the phase diagram of the IL. Upon loading the IL with Nd at ratio 4.45:1, the UCST of [Hbet][Tf_2_N] reduced from 55 °C to 35.1 °C and the mutual solubility of the biphasic system increased. Moreover, the presence of acid can affect significantly the dissolution of IL to aqueous phase. Mazan et al. found that the ILs solubility expectedly grows with the increase of the aqueous phase acidity [7]. In the studies of Volia et al., were the speciation of [Hbet][Tf_2_N] in hydrochloric media was investigated, it was found that the presence of acid increased the aqueous solubility of the IL cation while the effect of the IL anion was the opposite [8].

The main focus of this study was to investigate the most efficient method for Sc extraction from the ionic liquid leachate while maintaining the minimum IL losses in the aqueous strip solution. For the purpose of this study [Hbet][Tf_2_N] was used for leaching BR and a representative ionic liquid leachate was prepared. In order to investigate the process for metal extraction from the ionic liquid leachate, the latter was subjected in different stripping experiments using HCl as stripping agent at various volumes and concentrations. Both metal extraction efficiency and IL losses were considered for the evaluation of this method.

## 2. Materials and Methods

### 2.1. Materials

The IL [Hbet][Tf_2_N] was provided by IOLITEC with >97% purity and synthesis were confirmed with FTIR according to literature [12]. All the leaching experiments were conducted with BR provided by Mytillineos S.A. (Ag. Nickolas, Viotia, Greece). Samples of bauxite residue were characterized via a fusion method (1000 °C for 1 h with a mixture of Li_2_B_4_O_7_/KNO_3_ followed by direct dissolution in 6.5% HNO_3_ solution).

### 2.2. Preparation of Pregnant Ionic Liquid Solution (IL-PLS)

For the investigation of the metal extraction process from the IL [Hbet][Tf_2_N] to an acidic solution, a representative leachate solution (IL-PLS) was prepared by leaching BR using a [Hbet][Tf_2_N]-H_2_O mixture at conditions chosen by our previous reports [12] (40%wt.H_2_O addition, 10% pulp density, 90 °C, 4 h retention time).

For the leaching procedure the required amount of [Hbet][Tf_2_N] and H_2_O were weighted directly into the glass reactor of 1L under mechanical stirring in order to produce adequate quantity of IL-PLS for stripping experiments. Pure [Hbet][Tf_2_N] is a solid or a really viscous liquid at room temperature so for the IL was weighted in order to obtain the preferred amount. The conversion to volume units was carried out when necessary, using the density value of [Hbet][Tf_2_N], which was determined in the laboratory and is consistent with the value found in the literature (ρ = 1.53 gr/cm^3^) [10]. The heat was adjusted to desired temperature using an immersed thermocouple and then BR was added to the reactor. After the leaching, the pulp was filtrated under vacuum, resulting in a monophasic IL-PLS and a solid residue. The residue was washed in a separated flask with alcohol in order to rinse the remaining IL. The metal content in IL-PLS was determined using AAS and ICP-OES. The final concentration of metals is presented in Table 1. Besides Sc, which is the target element in this study, Fe and Al were measured as well, as they are considered the basic metal impurities for this process. Moreover, La and Y extraction behavior is investigated as representative elements for light and heavy rare earth elements respectively, which are also present in the described process.

Even though BR was treated with a biphasic IL-water mixture, the obtained IL-PLS remained monophasic even after cooling below the UCST of [Hbet][Tf_2_N]. The monophasic IL-PLS at ambient temperature contains entrapped the whole amount of water added during the leaching mainly for two reasons: (a) due to high dissolution of metals the UCST temperature has decreased at levels lower than the ambient temperature and (b) water molecules are incorporated in the formed metal-betainium complexes [9].

### 2.3. Stripping Experiments

After the leaching process a large amount of metals dissolved into the [Hbet][Tf_2_N]-H_2_O mixture, producing an ionic liquid leachate, or pregnant ionic liquid solution (IL-PLS). Stripping experiments were conducted directly to the IL-PLS using an acidic solution, in order to extract the metals from the IL to a final aqueous solution for further processing, similarly with typical solvent extraction processes. In a typical striping experiment, IL-PLS was mixed with an acidic solution of a specific concentration prepared in advance. For every stripping experiment the acidic solution and IL-PLS was added to a glass separating funnel according to the desired phase volume ratio. The phase volume ratio, *R*, is defined as the volume of the acidic solution (*V_aq_*) divided by the volume of the IL-PLS (*V_IL_*), as shown in Equation (1).

(1) $$R=\frac{V_{aq}}{V_{IL}}$$

The mixture is stirred vigorously at ambient temperature for 3 min manually. After 24 h of settling in a separating funnel, the mixture is completely clear with discrete interphase, indicating a full phase separation. Finally, the IL phase and the aqueous phase are obtained and their masses and volumes are measured. Finally, a sample is taken from the aqueous phase for metal concentration determination using AAS and ICP-OES and total organic carbon determination. The total deviation of both measurements is estimated about 10% on the final measured concentration. The metal extraction efficiency (%E) of the stripping process was calculated according to Equation (2):

(2) $$\%E=100\;\times \;\frac{m_{\left[\mathrm{aq}\right]}}{m_{\left[\mathrm{IL}\right]}}$$

where m_[aq]_ is the mass of the metal ion in the final aqueous solution, calculated with the measured concentration and volume in the final solution and m_[IL]_ calculated from the initial IL-PLS accordingly.

### 2.4. Ionic Liquid Losses Determination

In order to measure the IL dissolved in the PLS aqueous phase the Total Organic Carbon (TOC), Direct TNT Method using a DR/2500 spectrophotometer (HACH, Loveland, CO, USA) was employed. The results were converted into IL concentration; for this method the average calculated measurement deviation is ±1.1 gr [Hbet][Tf_2_N] per 100 mL of PLS. The IL losses (%L) of the process were calculated according to Equation (3):

(3) $$\%L=100\;\times \;\frac{\left[\mathrm{Hbet}\right]{\left[{\mathrm{Tf}}_{2}N\right]}_{\mathrm{final},\mathrm{aq}}}{\left[\mathrm{Hbet}\right]{\left[{\mathrm{Tf}}_{2}N\right]}_{\mathrm{initial}}}$$

where [Hbet][Tf_2_N]_final,aq_ is the mass of IL calculated in the aqueous phase after the stripping experiment and [Hbet][Tf_2_N]_initial_ is the total mass of IL introduced to the stripping process.

## 3. Results and Discussion

### 3.1. Metal Extraction from [Hbet][Tf_2_N] to HCl Acid Phase

During stripping procedure, the metals dissolved in the IL leachate are extracted with an acidic solution. The metal extraction mechanism can be described by a simple hydrogen-metal-ion exchange reaction shown in Equation (4). Where metals are extracted to the aqueous phase and the IL is regenerated:

(4) $$[M^{+x}{\left(\mathrm{bet}\right)}_{x\;}]\;{\left[{\mathrm{Tf}}_{2}N\right]}_{x\left(\mathrm{IL}\right)\;}+{\mathrm{xHCl}}_{\left(\mathrm{aq}\right)}\;\to {\mathrm{MCl}}_{x\left(\mathrm{aq}\right)}+{\mathrm{xHbetTf}}_{2}N_{\left(\mathrm{IL}\right)}$$

**Figure 2:**
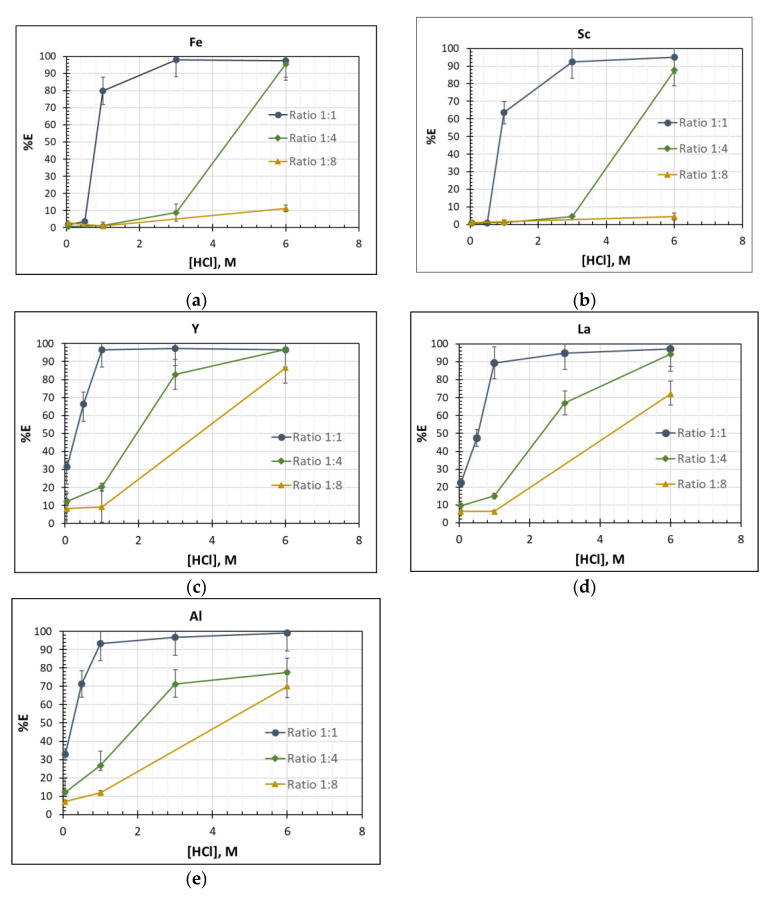
Extraction of each metal of interest as a function of HCl concentration in three different volume Ratios of acid:IL-PLS: (**a**) Fe, (**b**) Sc, (**c**) Y, (**d**) La, (**e**) Al.

In Equation (4) x is the oxidation number of the individual metal ions i.e., Fe^3+^, REE^3+^, Al^3+^ etc. whereas (bet) is the neutral zwitterion or deprotonated cation of [Hbet][Tf_2_N]. In Figure 2 it is demonstrated the metal extraction from the IL-PLS using HCl solutions in different volumetric ratios acid: IL-PLS and different concentrations of acid.

**Figure 3:**
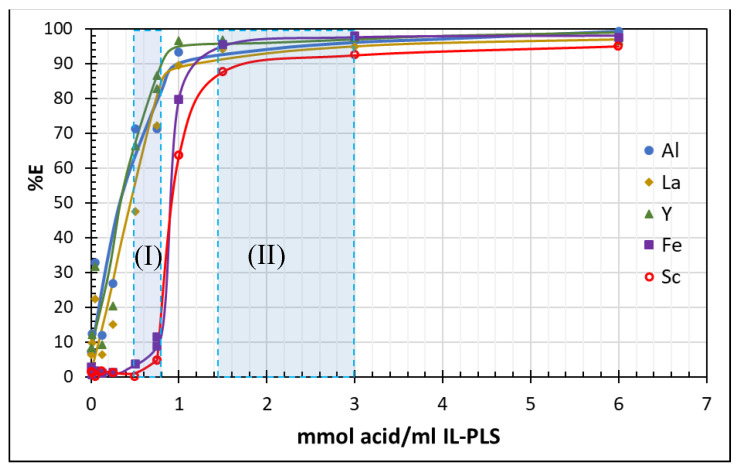
Extraction of metals as a function of the stoichiometric addition of HCl acid.Area (I) selective extraction of metals, area (II) complete extraction of metals.

As it is shown in Figure 2, the metal extraction increases as the concentration of the HCl increases for every metal in the three volume ratios tested. It is observed that metals can be divided in two different groups with similar extraction behavior Al, Y, La and Fe, Sc. In Figure 3 the metal extraction of the stripping experiments described before is expressed as a function of the stoichiometric addition of hydrochloric acid.

It is demonstrated in Figure 3 that the efficiency of the metal extraction is determined by the stoichiometric addition of acid in relation to the volume of the IL leach solution (IL-PLS). As a guide for the eye, a sigmoidal-like curve fits well to the observed behavior of Fe and Sc extraction, whereas Al, La and Y present a similar steep extraction curve.

From Figure 3 two distinct areas of interest can be observed in the metal extraction and these areas have been shaded as (I) and (II). In particular, in area (II) it is observed that with addition of more than 1.5 mmol of acid/mL IL-PLS, complete extraction of all metals can be achieved. At lower addition of acid 0.5–0.75 mmol HCl/mL IL-PLS-area (I), a selective extraction of metals is observed with high recoveries of Al, La and Y and very low recoveries of Fe and Sc. The latter two extracted from the IL-PLS at higher HCl addition, following a similar trend in their extraction, confirming their high chemical affinity and stronger complexation with the IL.

### 3.2. [Hbet][Tf_2_N] Dissolution in Aqueous Phase

In this section we describe the losses of the IL [Hbet][Tf_2_N] into the final aqueous solution after the same stripping experiments described in the previous section.

**Figure 4:**
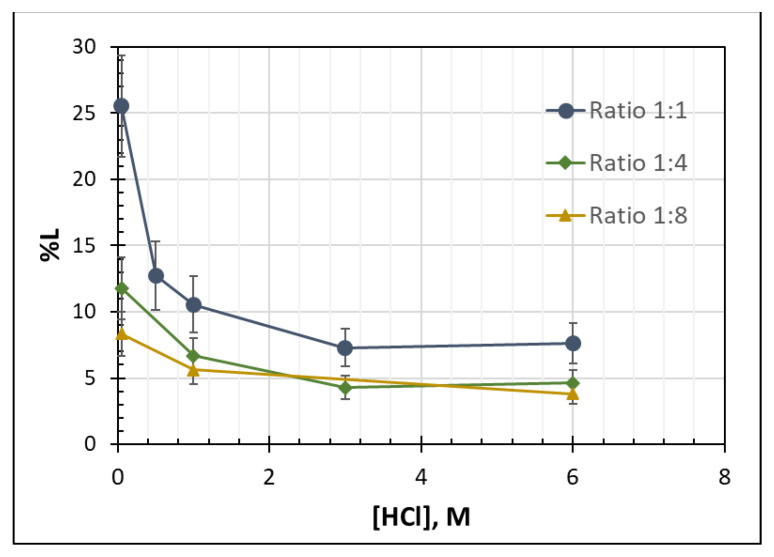
Losses % (%L) of ionic liquid [Hbet][Tf_2_N] in the final aqueous stripping solution.

In Figure 4, it is shown that the losses of the IL are increased when the volume ratio acid:IL-PLS is increased. That is to say, IL losses are higher when higher volumes of the acidic solution are introduced to the stripping process. The increase of the acidic solution volume inevitably leads to biphasic mixtures with higher quantity of aqueous phases, so the absolute amount of IL dissolved in the aqueous phase increases as well.

Moreover, under the same acid:IL-PLS volume ratio the IL losses decrease by increasing the acidic concentration of the strip solution. This is attributed to a pH-dependent behavior that [Hbet][Tf_2_N] exhibits [10]. When [Hbet][Tf_2_N] is treated with alkali solution, forms homogenous mixtures with water, despite the existing temperature. The acidification of the mixture leads to separation of the IL and the aqueous phase forming again a biphasic system. Therefore, the increased concentration of HCl acid in strip solution acidifies the system promoting the separation of IL and aqueous phase and thus decreasing the IL losses.

### 3.3. Developed Senarios for Stripping Process

Concerning the dissolution of the IL in the aqueous phase during direct acidic stripping, the minimum IL losses are achieved by decreasing the volume of the acid added in the process at the higher acidic concentration, as it is illustrated in Figure 4. In addition to this, it was found that the stoichiometric addition of the acid in the stripping process defines the metal extraction yields. Thus, at a constant stoichiometric addition, by reducing the volume ratios and increasing the acidic solution concentration accordingly, the same extraction yields can be achieved. Based on the metal extraction behavior, as well as the IL losses in the aqueous phase described in the previous sections, possible stripping procedures are examined. Two different extraction methods can be considered: a non-selective, one-stage extraction process and a two-stages process which is selective.

#### 3.3.1. One-Stage Non-Selective Stripping Process

For the one-stage extraction, amount of 1.5 mmol HCl/mL-IL PLS should be added at the minimum possible acid:IL-PLS volume ratio in order to achieve maximum extraction with minimum losses. Upon the first contact of the IL-PLS that has entrapped a big amount of water with an acidic solution water transfer is observed from the IL phase to the aqueous phase, due to the pH change. Regardless of the minimization in acidic volume addition, the significant increase in the aqueous phase reduces the final concentration in Sc even if the extraction rate achieved is high (see Table 2).

#### 3.3.2. Two-Stages Selective Stripping Process

Regarding the two-stages extraction, it was found that the use of hydrochloric acid exhibits a selective behavior in metals extraction. A stripping processes can be applied to the IL-PLS in order to separate the metals. With the addition of 0.5–0.75 mmol HCl/mL IL-PLS, Al and REEs can be extracted at high yields while Fe and Sc are not extracted. That is to say, a first step can be applied to separate Al and REEs from the IL-PLS and with a second step the remaining Sc and Fe can be extracted in a separate aqueous solution. Fe and Sc, as they have high chemical affinity, cannot be separated by each other through this process and should be separated in a further process. It must be noted that the consecutive stripping steps increase the IL losses in the aqueous phase, therefore it is important that the minimum volume of acid be added during the stripping processes.

In order to investigate the two-stages stripping process, IL-PLS was subjected to a first stripping step using 6 M HCl solution at a volume ratio 1:8 so that 0.75 mmols of HCl/mL IL-PLS to be added into the process. After the first step, the obtained IL phase was subjected in a second stripping step. Different concentrations of acidic solutions were examined by keeping constant the volume ratio acid:IL 1:8 during the second step.

**Figure 5:**
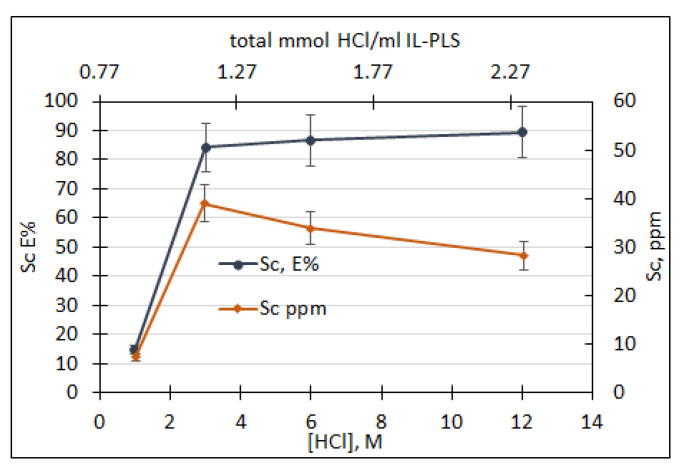
Scandium extraction and scandium concentration in aqueous phase during second stripping procedure as a function of the acid concentration and at constant volume ratio 1:8.

In Figure 5, it is observed that the increase of acid amount increases significantly the Sc extraction from 1 M HCl to 3M and above that concentration the change in extraction is negligible. In addition to this, it is shown in Figure 5 that the concentration of Sc is maximized at the concentration of 3 M while with the further increase of the acid addition decreases the Sc content to the aqueous phase. This observation can be attributed to the intense dissolution of IL into the aqueous phase that results in an increase of the overall volume of the aqueous phase.

Besides the metal extraction that occur during stripping process described in Equation (4), secondary reactions between the IL and the strip solution occur as well. In the beginning of the process the effect of IL-aqueous solution interaction is described mainly by the metals extraction reaction as the metals are extracted and the IL-metal complexes are depleted. Adding, excess of HCl acid results in an interaction in between the IL cation [Hbet]^+^ and the anion [Tf_2_Ν]^-^ with the chloride anions and the protons of HCl, increasing the dissolution of IL in the aqueous phase. This observation was not unexpected, as other reports in the literature state that increasing the acid increases the solubility of the IL in the aqueous phase [7,28,29]. Voila et al. described the dissolution mechanism of the IL [Hbet][Tf_2_N] in solutions of hydrochloric acid caused by dissociation species of the IL in the aqueous phase [8]. In the latter study it was found that higher acidic concentration increases the dissolution of the IL in the aqueous phase, mainly because the increased aqueous solubility of the IL cation.

**Figure 6:**
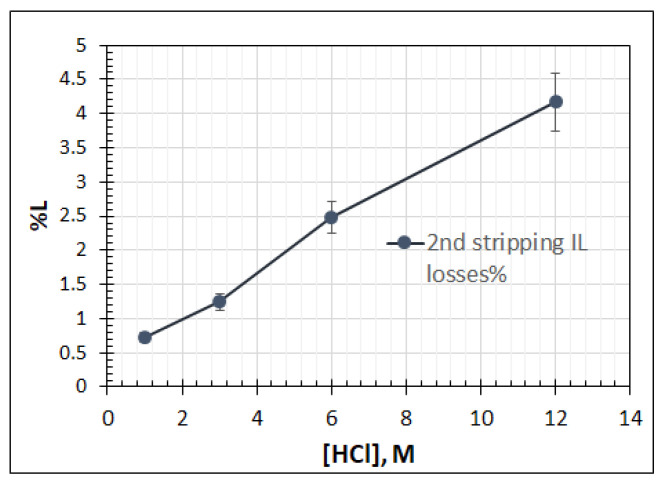
Ionic liquid losses % during the second stripping experiments.

This behavior is also supported by the measurements of IL losses during the second stripping step, illustrated in Figure 6, where the increase in the amount of acid increases the losses of IL, contrary to the observations of the initial stripping step.

#### 3.3.3. Evaluation of the Proposed Methods

**Figure 7:**
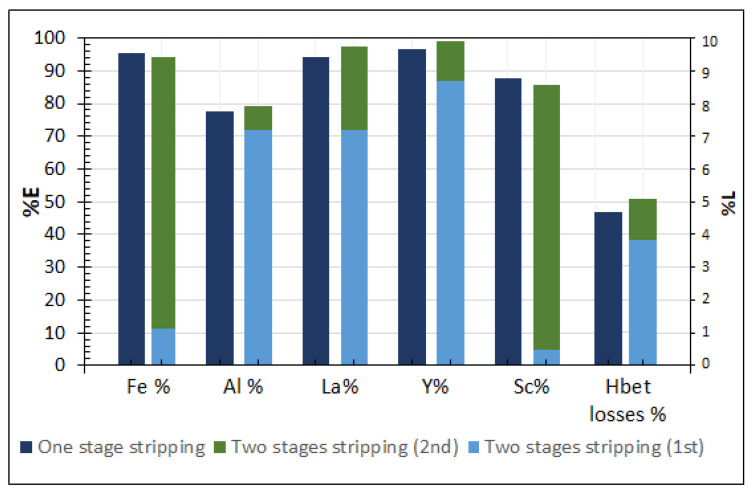
Comparison of the metal extraction and ionic liquid losses through the two different stripping approaches.

For evaluation of the methods proposed, Table 2 shows the concentration of the initial IL-PLS and the concentration of the produced aqueous streams, while in Figure 7 the extraction yields achieved through the too possible routes are compared.

From Table 2 and Figure 7 it is observed that with the two different routes proposed, similar extraction yields are achieved. The two-stage extraction presents slightly higher IL losses, attributed mainly in the first stripping step. Moreover, it is observed that with the two-stage process Sc is concentrated in the aqueous phase of the second stage reaching a concentration of 39 ppm which is 5 times higher than the one in the one stage stripping and 8 times higher than the one in the initial IL-PLS. This represents a significant overall improvement of the stripping test, when compared to our previously published results [11], where cumulative IL losses in the two-step stripping process where 17.5% and Sc concentration in the 2nd step strip solution was 26 ppm.

## 4. Conclusions

In this study the extraction of metals from the IL [Hbet][Tf_2_N] pregnant leaching solution were examined through an acidic stripping process. During the stripping different reactions occur simultaneously in the mixture. Besides the extraction of metals from the IL to the aqueous phase, water is transferred between the phases, changing the phase equilibrium, but at the same time part of the IL is also extracted into the aqueous phase.

The stripping process is a complex process, which can be affected by many factors, such as the acidity and the amount of strip solution added, the ratio of the phases as well as the initial state of the IL that is subjected to the process. Metals can be extracted at high yields to the aqueous solution through the acidic stripping process. It was found that the IL losses can be controlled by adjusting the acidity and the volume ratio of the phases during stripping. The different chemical affinity of metal cations like Sc and Fe with the IL, allows for designing impurity removal process, separating such cations from the other cations leached in the process. As presented in this work, the 4.7 ppm Sc concentration in the IL-PLS can be selectively increased to more than 8-fold in an aqueous PLS, while Fe concentration quadruples and other metal cations remain at similar or lower concentrations with the IL-PLS. At the same time the cumulative IL losses of this two-step process increase from 4.6% to only 5.1% when compared to the optimum single stripping step process. Furthermore approximately 80% of the IL losses happen during the first step of the stripping process where the bulk of the water content of the IL-PLS is stripped to the aqueous phase. Future work will focus on reclaiming or recycling this aqueous phase in the initial leaching step, thereby significantly reducing the total IL losses of the process.

## Figures and Tables

**Table 1:** Metal content concentration in the prepared IL-PLS.

	Fe (ppm)	Al (ppm)	La (ppm)	Y (ppm)	Sc (ppm)
**IL-PLS**	660.79	4450.85	2.06	4.07	4.73

**Table 2:** Comparison of the different concentration in metals in the different streams from the proposed stripping processes.

		mmol/mL IL-PLS	Fe (ppm)	Al (ppm)	La (ppm)	Y (ppm)	Sc (ppm)	IL Losses%
	**IL-PLS**		660.79	4450.85	2.06	4.07	4.73	
**One stage stripping**	6M HCl, R = 1:4	1.5	1220	6675	3.75	7.6	8	4.64
**Two stages stripping**	1st 6M HCl, R = 1:8	1.15	202	7600	4	9.5	0.594	5.07
	2nd 3M HCl, R = 1:8		5700	3300	5.3	5.9	39	

## Data Availability

The data presented in this study are available on request from the corresponding authors.

## Sample Availability

Samples of the compounds are not available from the authors.
